# A Medical Joke

**Published:** 1860-05

**Authors:** 


*A Medical Joke.*—The dead are never sick; consequently, all diseases may be classified as affections of the "Liver."

